# Magnetic resonance imaging of liver tumors using gadoxetic acid (Gd-EOB-DTPA) - pilot study

**DOI:** 10.1186/s12917-019-2038-y

**Published:** 2019-08-14

**Authors:** Pola Borusewicz, Ewa Stańczyk, Krzysztof Kubiak, Jolanta Spużak, Kamila Glińska-Suchocka, Marcin Jankowski, Piotr Sławuta, Dominika Kubiak-Nowak, Przemysław Podgórski

**Affiliations:** 1 Department of Internal Medicine and Clinic of Diseases of Horses, Dogs and Cats, Faculty of Veterinary Medicine Wroclaw, University of Environmental and Life Sciences, Wroclaw, Poland; 2 Center of Experimental Diagnostics and Innovative Biomedical Technology, Faculty of Veterinary Medicine, University of Environmental and Life Sciences, Wroclaw, Poland; 3 Clinic of Diagnostic Imaging, Vetsuisse Faculty, University of Zurich, Zurich, Switzerland; 4 Department and Clinic of Surgery, Faculty of Veterinary Medicine, University of Environmental and Life Sciences, Wroclaw, Poland; 5 Department of General and Interventional Radiology and Neuroradiology, Wroclaw Medical University, Wroclaw, Poland

**Keywords:** Liver tumors, MRI, Gadoxetic acid, Dogs, Heptobiliary-specific contrast media, Primovist®

## Abstract

**Background:**

Magnetic resonance imaging using gadoxetic acid, a hepatocyte-specific contrast agent, is one of the most useful MRI techniques used to diagnose liver tumours in humans. During the hepato-biliary phase, there is uptake of gadoxetic acid by normal hepatocytes, leading to hepatic parenchymal enhancement. This feature is used in human medicine to diagnose hepatic parenchymal metastatic disease, to differentiate primary liver tumours, to diagnose liver cirrhosis and focal nodular hyperplasia. This study presents the preliminary results of magnetic resonance imaging of focal lesions localised in the liver parenchyma in dogs following the administration of gadoxetic acid.

**Results:**

The lesion enhancement ratio (ER_lesion_) in the tumour metastasis was 0.05; the liver enhancement ratio (ER_liver_) – 0.49 and the post-contrast lesion-to-liver contrast ratio (CR) was 0.17. In dogs with hepatocellular hyperplasia, these values were 0.54; 0.51; and 1.18, respectively. In two dogs with a hepatic adenoma, the ER_lesion_ was 0.26 and 0.17, respectively; the ER_liver_ was 0.47 and 0.47, respectively and the CR was 0.33 and 0.31, respectively. In the dog with a neuroendocrine tumour, the ER_lesion_ was 0.03; the ER_liver_ amounted to 0.58 and the CR was 0.35. In the case of a hepatocellular carcinoma, these coefficients were 0.2, 0.6 and 0.3, respectively.

**Conclusion:**

Based on the results, it may be assumed that the MR images of the proliferative hepatic parenchymal lesions in dogs using gadoxetic acid are similar to those obtained in humans. This suggests that the contrast enhancement patterns used in human medicine may be useful in differentiating hepatic parenchymal lesions in dogs.

## Background

Proliferative hepatic lesions may be benign or malignant. Malignant lesions include primary tumours and hepatic parenchymal metastasis [1]. Primary liver tumours in dogs are rare and comprise 0.6–1.5% of all neoplastic lesions [2]. The most common of these include the hepatocellular carcinoma, the bile duct carcinoma as well as carcinoid and other primary epithelial tumours of the liver. Leiomyosarcomas and fibrosarcomas are much less common [3]. Hepatic metastases are twice as common, and the primary malignancies usually include primary neoplasia of the spleen, pancreas, and gastrointestinal tract [2]. Benign tumours of the liver parenchyma include focal nodular hyperplasia (FNH) and regenerative hepatocellular hyperplasia [1].

Due to the variety of lesions present in the liver parenchyma, new methods enabling their fast and non-invasive differentiation are currently sought. Presently, the most common methods used to diagnose proliferative hepatic parenchymal lesions include the ultrasound and computed tomography, which have low specificity [4, 5]. In human medicine, magnetic resonance imaging (MRI), combined with hepatocyte-specific contrast agents [6, 7] is the examination of choice for the differentiation of nodular parenchymal lesions in the liver [8].

Gadoxetic acid [(GD-EOB-DTPA), Primovist® other brand name Eovist®; Bayer-Schering Pharma, Germany] is a paramagnetic, hydrophilic, ionic contrast agent [6]. Following the arterial and venous phase, this contrast agent is taken up by functioning hepatocytes [9]. This feature is used in the diagnosis of liver parenchymal metastases in humans, to differentiate primary hepatic tumours, to diagnose liver cirrhosis and focal nodular hyperplasia (FNH) [6, 10–12]. There are few reports of the use of Gd-EOB-DTPA to study proliferative hepatic lesions in dogs. Different types of tumours have been analysed on a low-field MRI scanner [13], while remaining studies using Gd-EOB-DTPA were carried out only in the case of a hepatocellular carcinoma [14].

This study presents the preliminary results of MRI in dogs in the hepatobiliary phase following the administration of Gd-EOB-DTPA, a hepatocyte-specific contrast agent, using a high-filed MRI scanner to diagnose various types of focal lesions located in the liver parenchyma.

## Results

All the dogs qualified for general anaesthesia essential for MR imaging based on the results of the clinical and blood examination. Coagulation disorders, which are contraindications to core-needle biopsies and surgical tumour resection, were excluded in all the dogs. The abdominal ultrasound examination in all the dogs confirmed the presence of a proliferative lesion in the liver parenchyma. MRI was carried out in all the dogs, and the results of subjective assessment are presented in Table 1.

**Table 1:** The signal intensity changes in the lesion compared to the unchanged liver parenchyma in contrast sequences

Case		Pre-contrast		Post-contrast
		T1-W	T2-W	T1-W
I	tumour parenchyma	Hypointense relative to the surrounding parenchyma (+++)	Hyperintense relative to the surrounding parenchyma (+++)	No signal enhancement, hypointense compare to the surrounding parenchyma.
	central scar	–	–	–
	caverns	–	–	–
II	tumour parenchyma	Isointense relative to the surrounding parenchyma, heterogenous	Isointense relative to the surrounding parenchyma, heterogenous	Post-contrast signal enhancement, Isointense relative to the surrounding parenchyma
	central scar	Hypointense	Hyperintense	Hypointense
	caverns	–	–	–
III	tumour parenchyma	Moderately hypointense compared to the surrounding parenchyma	Moderately hyperintense compared to the surrounding parenchyma	Shows mild kontrast enhancement
	central scar	–	–	–
	caverns	Numerous, filled with a hypointense content	Numerous, filled with a hyperintense content	No contrast enhancement of content of caverns, strongly hypointense
IV	tumour parenchyma	Moderately hypointense compared to the surrounding parenchyma	Moderately hyperintense compared to the surrounding parenchyma	Showsmildcontrastenhancement
	central scar	–	–	–
	caverns	Numerous, filled with a hypointense content	Numerous, filled with a hyperintense content	No contrast enhancement of content of caverns, strongly hypointense
V	tumour parenchyma	Mildly hypointense	Moderately hyperintencewith a strongly hyperintense centre	No contrast enhancement, strongly hypointense
	central scar	–	–	–
	caverns	–	–	–
VI	tumour parenchyma	Heterogenous, moderately hypointense relative to the surrounding organ parenchyma	Heterogenous, moderately hyperintense relative to the surrounding organ parenchyma	Heterogenous, no contrast enhancement, strongly hypointense
	central scar	–	–	–
	caverns	Numerous, filled with a hypointense content	Numerous, filled with a hyperintense content	No contrast enhancement of content of caverns, strongly hypointense

**Fig. 1:**
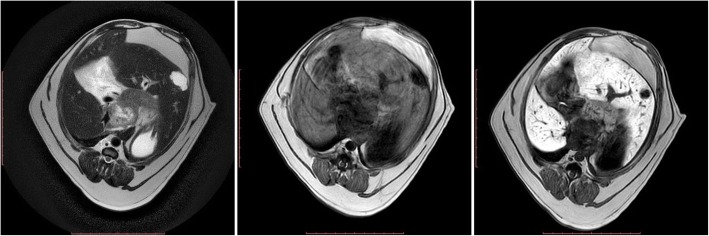
MRI of the liver parenchyma in dog I at the level of the proliferative lesion in T1-W images (left), T2-W images (centre) and T1-W-post-contrast images (right)

In the first dog, the abdominal ultrasound examination revealed the presence of a one small well defined, homogeneous space occupying lesion in the liver parenchyma, while MR imaging indicated the presence of two tumours. The first of these was oval with an irregular margin located in the lateral left hepatic lobe and measured 1.46 × 1.11 cm. The second lesion measured 2.28 × 1.43 cm and was visualised in the right lateral hepatic lobe as seen in the MRI. Both lesions were well demarcated and their signal intensity was similar in all sequences (Table 1) (Fig. 1). The lesion did not show contrast enhancement in the hepatobiliary phase. The normal liver parenchyma was homogenous. The histopathologic analysis of the biopsied sections revealed the presence of a metastatic carcinoma.

**Fig. 2:**
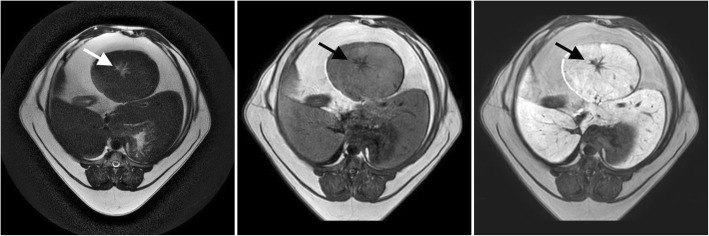
MRI of the liver parenchyma in dog II at the level of the proliferative lesion in T1-W images (left), T2-W images (centre) and T1-W-post-contrast images (right). A central scar was visible in the centre of the lesion (arrow)

In the second dog, a large hepatic space occupying lesion, most likely originating from the quadrate lobe, was diagnosed based on the ultrasound examination. The MR images confirmed the initial location of the lesion. In addition, enlargement of the lobe was noted, measuring 5.66 × 7.43 cm with irregular, rounded margins (Table 1) (Fig. 2). In the hepatobiliary phase, the lesion showed contrast enhancement similar to that of normal liver parenchyma. In the centre of the lesion, a central scar, which was hypointense, was observed. Based on the histopathologic analysis of the biopsy sections, hepatocellular hyperplasia/dysplasia was diagnosed.

**Fig. 3:**
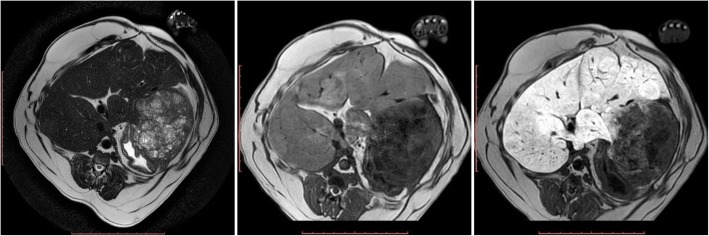
MRI of the liver parenchyma in dog III at the level of the proliferative lesion in T1-W images (left), T2-W images (centre) and T1-W-post-contrast images (right)

The third patient was diagnosed with a large lesion in the left hepatic lobe. The MRI confirmed a proliferative lesion measuring 9.38 × 6.33 cm, located in the left lateral hepatic lobe. The lesion was oval, well-demarcated with smooth margins (Table 1) (Fig. 3). The lesion showed moderate contrast enhancement in the hepatobiliary phase. Cysts, which did not show contrast enhancement, were present within the lesion. Due to the lack of owner consent to resect the lesion, a tru-cut needle biopsy was carried out. The histopathologic analysis of the sections indicated a hepatocellular adenoma.

**Fig. 4:**
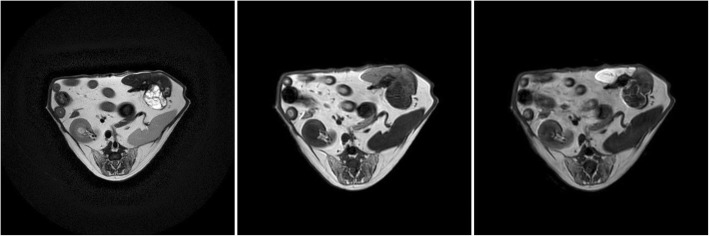
MRI of the liver parenchyma in dog IV at the level of the proliferative lesion in T1-W images (left), T2-W images (centre) and T1-W-post-contrast images (right)

The ultrasound examination in the fourth dog revealed the presence of a lesion in the left lateral hepatic lobe. MR imaging confirmed the presence of a clearly visible 3.81 × 3.04 cm lesion with irregular margins in the left lateral lobe (Table 1) (Fig. 4). The lesion showed moderate contrast enhancement in the hepatobiliary phase. Cysts, which did not show contrast enhancement, were present within the lesion. The dog was qualified for surgical resection of the lesion. A histopathologic assessment of the resected lesion indicated the presence of a hepatocellular adenoma.

**Fig. 5:**
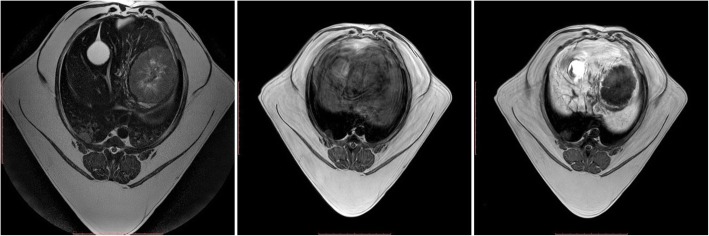
MRI of the liver parenchyma in dog V at the level of the proliferative lesion in T1-W images (left), T2-W images (centre) and T1-W-post-contrast images (right)

A lesion in the left hepatic lobe was diagnosed in the ultrasound examination of the fifth dog. MRI revealed that the entire medial segment of the left lateral lobe was mildly hyperintense compared to the remaining lobes. The lesion was 5.3 × 6.25 cm large and was located in close proximity to large blood vessels (Table 1) (Fig. 5). No parenchymal contrast enhancement was observed in the hepatobiliary phase. The lesion was strongly hypointense compared with the remaining organ parenchyma. The owners declined surgery due to its high risk and the age of the dog. The histopathologic assessment of the biopsy sections indicated a neuroendocrine tumour (carcinoid tumour).

**Fig. 6:**
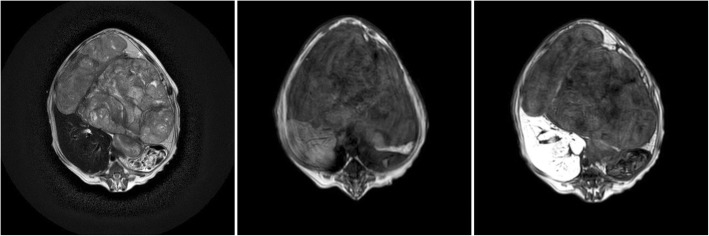
MRI of the liver parenchyma in dog VI at the level of the proliferative lesion in T1-W images (left), T2-W images (centre) and T1-W-post-contrast images (right)

In the sixth dog, a multiple large and small space occupying lesions were seen in the abdominal ultrasound; the lesions in the hepatic parenchyma were extensive and their exact localisation in a specific liver lobes was possible only based on MR imaging. The proliferative lesions were 0.5 to 10 cm large and were present in all the hepatic lobes (Table 1) (Fig. 6) except for the caudate lobe, which was unchanged. The lesion was heterogenous and strongly hypointense in the hepatobiliary phase and did not show contrast enhancement. The dog was euthanised and sections of the lesions were collected at necropsy. The histopathological analysis of the sections indicated the presence of a malignant hepatocellular carcinoma.

An analysis of the signal enhancement was carried out to confirm the subjective assessment of the MR images in all six dogs (Table 2).

**Table 2:** The signal intensity in pre- and post-contrast T1-W images of the parenchymal tumours in dogs

Case	Normal liver parenchyma		Lesion	
	Pre-contrast	Post-contrast		
			Pre-contrast	Post-contrast
I	924.08 (SD = 6.08)	1812.44 (SD = 335.74)	288.0 (SD = 38.02)	303.12 (SD = 29.31)
II	738.91 (SD = 16.78)	1510.0 (SD = 50.47)	829.11 (SD = 40.30)	1788.26 (SD = 94.15)
III	971.69 (SD = 24.33)	1852.40 (SD = 119.77)	455.78 (SD = 36.78)	616.11 (SD = 57.47)
IV	882.83 (SD = 17.59)	1666.05 (SD = 61.59)	430.92 (SD = 23.37)	521.72 (SD = 52.79)
V	622.42 (SD = 31.72)	1477.15 (SD = 88.06)	505.11 (SD = 71.38)	520.83 (SD = 60.35)
VI	749.55 (SD = 58.62)	1874.63 (SD = 44.16)	446.88 (SD = 32.37)	558.97 (SD = 22.47)

The ER _lesion_, ER _liver_ and CR for all the lesions are presented in Table 3.

**Table 3:** The enhancement ratios of the lesion and liver parenchyma and the post-contrast lesion-to-liver contrast ratio

Case	ER _lesion_	ER _liver_	CR
I	0.05	0.49	0.17
II	0.54	0.51	1.18
III	0.26	0.47	0.33
IV	0.17	0.47	0.31
V	0.03	0.58	0.35
VI	0.2	0.6	0.30

## Discussion

The presented study, which was carried out on a high-field MR scanner, enabled a preliminary comparison of various types of proliferative hepatic parenchymal lesions in dogs in the post-contrast examination. The data were recorded approximately 26 min after contrast administration. This is in accordance with own observations [15] and the observations of other authors, who suggest that post-contrast sequences should be obtained between 20 and 40 min after contrast administration [16].

The MRI appearance of the metastatic lesions observed in the first patient were similar to those observed in humans. In humans, liver metastases are typically multifocal and appear in the form of irregularly marginated parenchymal lesions with low signal intensity on T1 weighted images and are hyperintense on T2-weighted images in comparison to normal hepatic parenchyma. These lesions do not show contrast enhancement in the hepatobiliary phase [6, 17]. In the presented case, the ER_lesion_ was 0.05, which indicated that there was no signal enhancement in the post-contrast sequence. The CR in that dog was low and amounted to 0.17, consistent with a large difference in the signal intensity between the normal liver tissue and the parenchymal lesions. That is also in accordance with the findings in humans [6].

In the second dog, focal nodular hyperplasia (FNH) was diagnosed in the quadrate lobe based on a histopathologic examination of a biopsy section of the liver. The compromised lobe was homogenous with rounded margins, which corresponds to the description of this type of lesion in humans [11]. In human medicine, FNH appears as small homogenous well-demarcated lesions with no encapsulation [11]. In all the sequences, the parenchyma of the lesion in the examined dog was isointense compared to the surrounding normal liver parenchyma. In a study describing FNH in contrast-enhanced low-field MRI in dogs using gadoxetic acid [13], the authors reported that the lesions were hyperintense compared to the normal liver parenchyma in T2-W images and isointense in precontrast T1-W images. The parenchyma of the lesions enhanced contrast comparably or more than the normal liver parenchyma. In humans, FNH is usually moderately hypointense in pre-contrast T1-W images, moderately hyperintense in T2-W images and isointense or hyperintense in post-contrast T1-W images [11]. In some human studies, similarly to the reported dog in this study, FNH is isointense compared to the surrounding normal liver parenchyma in pre-contrast T1-W and T2-W images [18]. In the studied dog, a central scar was also present in all the sequences, which is a feature of most FNHs in humans [11, 18]. The scar in the described dog was hyperintense in T2-W images and hypointense in the pre- and postcontrast T1-W images. In human medicine, such characteristics of the central scar are considered typical of FNH and facilitate the differential diagnosis between FNH, adenoma and hepatocellular carcinoma (HCC). The MRI appearance of the latter two tumours may be similar to FNH. However, in the case of adenoma, presence of the central scar is not reported, while the central scar in HCC is hypointense in all sequences [18]. In humans,the central scar in the course of FNH is present in 25% of patients in T2-W images and is usually hyperintense. The central scar is also present in pre- and post-contrast T1-W images in 36% of the patients [19]. In their study, Yonetomi et al. [13] did not report the presence of a central scar in four dogs with FNH. The imaging findings of FNH in the reported dog is consistent with the findings in humans with the same disease. The subjective assessment of the signal intensity of the liver parenchyma and the lesion was confirmed based on the calculations of the CR, which amounted to 1.18, indicating that the normal liver tissue and the lesion underwent similar contrast enhancement. This is also confirmed by the comparable values of the ER_lesion_ and ER_liver_ in this dog.

The third and fourth dog were diagnosed with a hepatocellular adenoma based on the histopathologic assessment of biopsy section. In both cases, there was a single tumour in the liver parenchyma, which was significantly different in size. In the third dog, the tumour was well delineated from the surrounding liver parenchyma, while in the fourth dog it was ill defined in pre-contrast sequences. Both lesions were mildly hypointense compared to the surrounding liver parenchyma on pre-contrast T1-W images and mildly hyperintense on T2-W images. In humans, similarly to the presented dog, hepatocellular adenomas are usually single lesions. However, they are usually hyperintense to isointense on pre-contrast T1-W images compared to the surrounding organ parenchyma [18, 19]. Another study reported that up to 36% of adenomas in humans are hypointense on T1-W pre-contrast images, which is consistent with the findings in this study [20]. In humans, the lesions are also reported to be slightly hyperintense on T2-W images, which is in accordance with the present findings in the two dogs. In both dogs, there were caverns, filled with content which appeared strongly hypointense on T1-W images and strongly hyperintense on T2-W images. These lesions may indicate necrotic foci within the tumour [18]. Such findings were also reported in adenomas in human patients. The presence of lipid depositions and calcification within the lesions have also been reported in humans, but were not observed in the studied dogs [18]. This may be due to the fact that there are many types of adenomas in humans, such as inflammatory hepatocellular adenoma, HNF-1α-mutated hepatocellular adenoma or the β- catetin- mutated hepatocellular adenoma, not distinguished in dogs. In both dogs diagnosed with a hepatocellular adenoma, there was partial weak contrast-enhancement of the lesion parenchyma, and the CR was similar in both cases and amounted to 0.33 and 0.31, respectively. These values were lower than those in the dog with FNH (case no. 2) and higher than those reported in the dog with liver metastases (case no. 1).

A neuroendocrine hepatic tumour (carcinoid) was diagnosed in the fifth case based on a histopathologic analysis of the biopsy sections. This tumour is rarely described in humans and animals [21–23]. There are no reports of a MR examination in a dog with a primary hepatic carcinoid tumour. There is a single description of MR imaging using Gd-EOB-DTPA, a hepatocyte-specific contrast agent in the case of a primary hepatic carcinoid tumour in a human [23]. In the studied dog, the tumour was heterogenous and slightly hypointense compared to the surrounding liver parenchyma, heterogenous, while the centre of the tumour was strongly hypointense. This does not correspond to the pre-contrast T1-W images obtained in humans, where the tumour was weakly hyperintense. The tumour parenchyma was hyperintense compared to the surrounding parenchyma in the T2-W sequence, and there was a stellar hyperintense area in the central part of the lesion. A similar finding was reported by Baek et al. [23], who found that the tumour was hyperintense in T2-W images compared to the surrounding liver parenchyma. Those authors also found that there were strongly hyperintense foci within the liver parenchyma, which remained hypointense in T1-W images. Baek et al. [23] interpreted those foci as a haemorrhagic component. Such foci were not observed in the studied dog. In the reported dog, there was no lesion contrast enhancement in post-contrast T1-W images, which is consistent with the findings in humans [23]. Baek et al. [23] are the first authors to characterise a primary hepatic carcinoid tumour in a human using gadoxetic acid. Hence, it is unclear whether there are other possible contrast enhancement patterns in this tumour. In the reported dog, the ER_lesion_ measuring 0.03 confirmed that there was no contrast enhancement within the lesion, while the CR was 0.35 and was similar to the CR of the dog with the liver adenoma.

In the sixth dog, a hepatocellular carcinoma was diagnosed based on the histopathologic examination of the liver biopsy sections. This is the most common primary hepatic tumour in dogs [1, 2, 14]. In the studied dog, the findings on the pre- and post-contrast MR-images are consistent with the reports of other authors [13, 14]. The lesions were heterogenous, and were hypoechogenic in pre-contrast T1-W sequences, which, according to Constanst et al. [14] is observed in 12.5% canine patients with hepatocellular carcinoma. In humans with hepatocellular carcinomas, the lesions appear isointense compared to the surrounding organ parenchyma on pre-contrast T1-W images, but some lesions may appear hyperintense or hypointense with hyperintense regions [24]. The lesions in the studied dog were also heterogenous and hyperintense on T2-W images compared to the organ parenchyma. This is consistent with the reports of this type of tumour in humans [18]. However, in another study carried out on dogs, the lesions were reported to be hyperintense relative to the surrounding parenchyma in only 12.5% of the cases [14]. In the study carried out on dogs, Constant et al. [14] found that there was no lesion contrast enhancement in post-contrast sequences [14], which was also observed in the present study. In humans, such lesions have low signal intensity on post-contrast images. It was also found that the degree of heterogeneity is variable and depends on the concentration and function of cellular membrane receptors and transporters [18]. The differences in the lesion heterogeneity between the dog and reports of heterogeneity in humans may be attributed to the differences in tumour size between species. In humans, the tumours are usually diagnosed at an earlier stage [25]. In contrast, the tumour in the studied dog was terminal and affected the majority of the liver parenchyma. This led to the formation of numerous necrotic foci present on MRI as caverns filled with a T1-W hypointense and T2-W hyperintense content. The ER_lesion_ was 0.2 indicating that there was moderate contrast enhancement, while the CR was similar to that obtained in the case of the hepatic adenoma and neuroendocrine tumour.

## Conclusions

The magnetic resonance imaging findings of proliferative lesions in the liver parenchyma in dogs using Gd-EOB-DTPA, a hepatocyte-specific contrast agent, are similar to those reported in humans with the same types of lesions. This finding warrants further investigation on a larger population of dogs.

### Limitations

One limitation of the study was a small and heterogenous study group; hence only preliminary conclusions regarding the post-contrast imaging findings of proliferative hepatic lesions could be drawn. Another limitation was the lack of an assessment of the early (arterial and venous) contrast phase, which may have enabled a more detailed differential diagnosis of the lesions. The authors plan to study post-contrast imaging findings of hepatic lesions on a larger group of dogs. They also plan to research all the contrast enhancement stages in liver disease.

## Methods

In accordance with Art. 1 point 2.1 to the Act from January 15th 2015 on the Protection of Animals Used for Scientific or Educational Purposes, the study and all associated procedures do not require the approval of the local ethics committee.

The study was carried out on six dogs of different breeds (2 fox terriers, 2 mixed-breed dogs, 1 miniature schnauzer, 1 longhaired Dachsund) of both sexes (4 females, 2males), who were from 10 to 13 years old (mean = 11.83, SD = 1.17). All the dogs were patients of the outpatient clinic of the Department of Internal Medicine and Clinic of Diseases of Horses, Dogs and Cats of the Faculty of Veterinary Medicine at the Wroclaw University of Environmental and Life Sciences. Focal lesions in the liver parenchyma were identified during routine abdominal ultrasound examinations in all cases. A clinical examination and laboratory blood tests (complete blood count, biochemical parameters, coagulogram) were carried out prior to MRI to determine whether the animals qualified for further imaging and a core needle biopsy. The animals were premedicated with medetomidine administered at 0.005 mg/kg i.m. (Narcostart 1 mg/ml, Le Vet B.V.,Oudewater, Holandia) and butorphanol at 0.005 mg/kg i.m. (Torbugestic 10 mg/ml, Pfizer, Warszawa, Polska). General anaesthesia was induced with propofol administered at 1 mg/kg i.v. (Scanofol 10 mg/ml, ScanVet, Skiereszewo, Polska). Anaesthesia was maintained with isoflurane (Forane 100%, AbbVie, Warszawa, Polska). The respiratory rate, heart rate, oxygen saturation, carbon dioxide concentrations in the respired gases and the inspiratory and expiratory concentration of the anaesthetic mixture were monitored during the MRI examination. MRI was performed on a 1.5-T MRI system (Ingenia; Philips Healthcare, Best, The Netherlands) using a 32-channel torso phased array coil. All sequences were carried out with respiratory-triggering. Dogs were positioned in dorsal recumbence. The MRI protocol included the T1-W TFE and T2-W TSE sequences in the transverse plane. Subsequently, Gd-EOB-DTPA (Primovist®), a hepatocyte-specific contrast agent was administered intravenously at 0.1 ml/kg (0.025 mmol/kg), followed by 15 ml of a 0.9% saline solution. The post-contrast T1-W TFE sequence was acquired 26 min after contrast administration, in the transverse plane (Table 4). The MRI were assessed by two independent radiologists (PB and ES), one of whom was an ECVDI diplomate.

**Table 4:** MR sequences and imaging parameters used during the study

	Pre-contrast T1-W TFE	T2-W MV HR	Post-contrast T1-W TFE
Echo time (ms)	4.6	100	4.6
Repetition time (ms)	10	3745	10
Flip angle (°)	25	90	25
Bandwidth (Hz)	141.7	531.9	141.7
FOV (mm)	220 × 192	250 × 250	220 × 192
Matrix	220 × 192	500 × 500	220 × 192
Slice thickness (mm)	3	5	3
GAP (mm)	0	0.5	0
Number of slices	100	50	100

Based on the abdominal ultrasound and liver MR images, one of the dogs was qualified for a surgical resection of the liver tumour. Four of the studied dogs were eligible for a core-needle biopsy of the hepatic parenchymal lesion, while the owners of one dog requested euthanasia. Liver tumour samples were collected from that dog at necropsy. The core-needle biopsy was carried out using a 16G tru-cut needle. The biopsy was obtained from the marginal area of the lesion. The samples then underwent histopathologic evaluation.

The obtained MR images were analysed using the Alteris Osirix 1.5.8 (Alteris S.A., Poland) software. The T1 and T1 post contrast signal intensity of the tumour was compared to the unchanged liver parenchyma, the homogeneity of the lesion, its size, extent and localisation were also analysed. In the T1 pre and post contrast studies the oval regions of interest (ROI) were set manually in chosen areas to assess the signal enhancement of the liver parenchyma and the tumour. Three ROIs were localised within the unchanged liver parenchyma, and three were placed within the tumour. Where possible, the ROIs were localised away from large blood vessels.

Based on the obtained results of the signal intensity in the liver parenchyma for each ROI, an arithmetic mean of the signal intensity of the liver parenchyma before (SI _liverpre-contrast_) and after (SI _liverpost-contrast_) contrast administration as well as the tumour signal intensity before (SI _tumor pre-contrast_) and after (SI _tumor post-contrast_) contrast injection were calculated. Based on those mean values, the enhancement ratios (ER) and the post-contrast lesion-to-liver contrast ratio (CR) were calculated using the following formulae [26, 27]:

$$ {\mathrm{ER}}_{\mathrm{lesion}}=\left({\mathrm{SI}}_{\mathrm{tumor}\ \mathrm{post}-\mathrm{contrast}}-{\mathrm{SI}}_{\mathrm{tumor}\ \mathrm{pre}-\mathrm{contrast}}\right)/{\mathrm{SI}}_{\mathrm{tumor}\ \mathrm{post}-\mathrm{contrast}}{\mathrm{ER}}_{\mathrm{liver}}=\left({\mathrm{SI}}_{\mathrm{liver}\ \mathrm{post}-\mathrm{contrast}}-{\mathrm{SI}}_{\mathrm{liver}\ \mathrm{pre}-\mathrm{contrast}}\right)/{\mathrm{SI}}_{\mathrm{liver}\ \mathrm{post}-\mathrm{contrast}}\mathrm{CR}={\mathrm{SI}}_{\mathrm{tumor}\ \mathrm{post}-\mathrm{contrast}}/{\mathrm{SI}}_{\mathrm{liver}\ \mathrm{post}-\mathrm{contrast}} $$

## Data Availability

The datasets used and/or analysed during the current study are available from the corresponding author on reasonable request.

## Abbreviations

CR: Contrast ratio
ER: Enhancement ratio
FNH: Focal nodular hyperplasia
FOV: Field of view
G: Gauge
Gd-EOB-DTPA: Gadoxetic acid
HCC: Hepatocellular carcinoma
HNF: Hepatocyte nuclear factor
HR: High resolution
MR: Magnetic resonance
MRI: Magnetic resonance imaging
MV: MultiVane
ROI: Region of interest
SD: Standard deviation
SI: Signal intensity
T1-W: T1- weighted
T2-W: T2 - weighted
TFE: Turbo field echo
TSE: Turbo spin echo

## References

[1] Cullen JM, Popp JA. Tumors of the liver and gall bladder. In: Meuten DJ, editor. Tumors in Domestic Animals. Iowa State Press; 2002. pp 483–508.

[2] Liptak JM, Dernell WS, Withrow SJ (2004). Liver tumors in cats and dogs. Compend Contin Educ Pract Vet.

[3] Patnaik AK, Hurvitz AI, Lieberman PH (1980). Canine hepatic neoplasms: a clinicopathologic study. Vet Pathol.

[4] Lamb CR (1990). Abdominal ultrasonography in small animals*:* examination of the liver, spleen and pancreas. J Small AnimPract.

[5] Jones ID, Lamb CR, Drees R, Priestnall SL, Mantis P (2016). Associations between dual-phase computed tomography features and histopathologic diagnoses in 52 dogs with hepatic or splenic masses. Vet Radiol Ultrasound..

[6] Zech CJ, Herrmann KA, Reiser MF, Schoenberg SO (2007). MR imaging in patients with suspected liver metastases: value of liver-specific contrast agent Gd-EOB-DTPA. Magn Reson Medi Sci.

[7] Hammerstingl R, Huppertz A, Breuer J, Balzer T, Blakeborough A, Carter R, Fusté LC, Heinz-Peer G, Judmaier W, Laniado M, Manfredi RM, Mathieu DG, Müller D, Mortelè K, Reimer P, Reiser MF, Robinson PJ, Shamsi K, Strotzer M, Taupitz M, Tombach B, Valeri G, van Beers BE, Vogl TJ (2008). Diagnostic efficacy of gadoxetic acid (Primovist)-enhanced MRI and spiral CT for a therapeutic strategy: comparison with intraoperative and histopathologic findings in focal liver lesions. Eur Radiol.

[8] Clifford CA, Pretorius ES, Weisse C, Sorenmo KU, Drobatz KJ, Siegelman ES, Solomon JA (2004). Magnetic resonance imaging of focal splenic and hepatic lesions in the dog. J Vet Intern Med.

[9] Hamm B, Staks T, Mühler A, Bollow M, Taupitz M, Frenzel T, Wolf KJ, Weinmann HJ, Lange L (1995). Phase I clinical evaluation of Gd-EOB-DTPA as a hepatobiliary MR contrast agent: safety, pharmacokinetics, and MR imaging. Radiology..

[10] Frericks BB, Loddenkemper C, Huppertz A, Valdeig S, Stroux A, Seja M, Wolf KJ, Albrecht T (2009). Qualitative and quantitative evaluation of hepatocellular carcinoma and cirrhotic liver enhancement using Gd-EOB-DTPA. Am J Roentgenol.

[11] Zech CJ, Grazioli L, Breuer J, Reiser MF, Schoenberg SO (2008). Diagnostic performance and description of morphological features of focal nodular hyperplasia in Gd-EOB-DTPA-enhanced liver magnetic resonance imaging: results of a multicenter trial. Investig Radiol.

[12] Van Kessel CS, De Boer E, Ten Kate FJW, Brosens LAA, Veldhuis WB, Van Leeuwen MS (2013). Focal nodular hyperplasia: hepatobiliary enhancement patterns on gadoxetic-acid contrast-enhanced MRI. Abdom Imaging.

[13] Yonetomi D, Kadosawa T, Miyoshi K, Nakao Y, Homma E, Hanazono K, Yamada E, Nakamura K, Ijiri A, Minegishi N, Maetani S, Hirayama K, Taniyama H, Nakede T (2012). Contrast agent Gd-EOB-DTPA (EOB·Primovist®) for low-field magnetic resonance imaging of canine focal liver lesions. Vet Radiol Ultrasound.

[14] Constant C, Hecht S, Craig LE, Lux CN, Cannon CM, Conklin GA (2016). Gadoxetate disodium (Gd-EOB-DTPA) contrast enhanced magnetic resonance imaging characteristics of hepatocellular carcinoma in dogs. Vet Radiol Ultrasound..

[15] Borusewicz P, Stańczyk E, Kubiak K, Spużak J, Glińska-Suchocka K, Jankowski M, Nicpoń J, Podgórski P (2018). Liver enhancement in healthy dogs after gadoxetic acid administration during dynamic contrast-enhanced magnetic resonance imaging. Vet J.

[16] Marks AL, Hecht S, Stokes JE, Conklin CA, Deanna KH (2014). Effects of Gadoxetate disodium (Eovist®) contrast on magnetic resonance imaging characteristics of the liver in clinically healthy dogs. Vet Radiol Ultrasound..

[17] Grazioli L, Morana G, Federle MP (2001). BrancatelliG, Testoni M, Kirchin MA, Olivetti L, Nicoli N, Procacci C. focal nodular hyperplasia: morphologic and functional information from MR imaging with GadobenateDimeglumine. Radiology..

[18] Campos JT, Sirlin CB, Choi JY (2013). Focal hepatic lesions in Gd-EOB-DTPA enhanced MRI: the atlas. Insights Imaging.

[19] Grazioli L, Bondioni MP, Haradome H, Motosugi U, Tinti R, Frittoli B, Gambarini S, Donato F, Colagrande S (2012). Hepatocellular adenoma and focal nodular hyperplasia: value of gadoxetic acid–enhanced MR imaging in differential diagnosis. Radiology..

[20] Grieser C, Steffen IG, Kramme IB, Bläker H, Kilic E, Perez Fernandez CM, Seehofer D, Schott E, Hamm B, Denecke T (2014). Gadoxetic acid enhanced MRI for differentiation of FNH and HCA: a single Centre experience. Eur Radiol.

[21] Patnaik AK, Lieberman PH, Hurvitz AI, Johnson GF (1981). Canine hepatic carcinoids. Vet Pathol.

[22] Patnaik AK, Newman SJ (2005). Scase T, ErlandsonRA, Antonescu C, craft D, Bergman PJ. Canine hepatic neuroendocrine carcinoma: an immunohistochemical and electron microscopic study. Vet Pathol.

[23] Baek SH, Yoon JH, Kim KW (2013). Primary hepatic neuroendocrine tumor: gadoxetic acid (Gd-EOB-DTPA) - enhanced magnetic resonance imaging. Acta Radiol Short Rep.

[24] Levy AD (2002). Malignant liver tumours. Clin Liver Dis.

[25] Balogh J, Victor D, Asham EH, Burroughs SG, Boktour M, Saharia A, Li X, Ghobrial RM, Monsour HP (2016). Hepatocellular carcinoma: a review. J Hepatocell Carcinoma.

[26] Narita M, Hatano E, Arizono S, Miyagawa-Hayashino A, Isoda H, Kitamura K, Taura K, Yasuchika K, Nitta T, Ikai I, Uemoto S (2009). Expression of OATP1B3 determines uptake of Gd-EOB-DTPA in hepatocellular carcinoma. J Gastroenterol.

[27] Ni Y, Chen F, Wang H, Feng Y, Junjie L, Jiang Y (2010). Proper definitions of MRI contrast enhancement in liver tumors. J Gastroenterol.

